# Exploring the Impact of the COVID-19 Pandemic on Student Mental Health: A Repeated Cross-Sectional Study

**DOI:** 10.3390/ijerph22060913

**Published:** 2025-06-08

**Authors:** Joanne Worsley, Jason McIntyre, Rhiannon Corcoran

**Affiliations:** 1 Department of Primary Care and Mental Health, University of Liverpool, Liverpool L69 7ZA, UK; corcoran@liverpool.ac.uk; 2 School of Psychology, Liverpool John Moores University, Liverpool L3 3AF, UK; j.c.mcintyre@ljmu.ac.uk

**Keywords:** COVID-19 pandemic, student mental health, depression, anxiety, eating disorders, repeated cross-sectional survey

## Abstract

**Background**: Although mental health among students has become a pressing public concern over recent years, the COVID-19 pandemic introduced new stressors, which may further increase the mental health burden for them. While past work has investigated links between pandemic related factors and student mental health and wellbeing, there is conflicting evidence around some symptoms (e.g., anxiety) and little work has focused on less common mental health conditions (e.g., eating disorders). **Aims**: The current study aimed to detail the prevalence of mental distress in the student population at an early stage of the COVID-19 pandemic and compare university students’ mental health before and during the COVID-19 pandemic. Specifically, we aimed to compare levels of depression, anxiety, and eating disorders in a large sample of students. **Methods**: We analysed data from a repeated cross-sectional survey on different samples of UK university students before the pandemic (*n* = 4812) and during the pandemic (*n* = 3817). **Results**: There were high levels of depression and anxiety during the COVID-19 pandemic, with more than 50% experiencing levels above the clinical cut-offs. Findings revealed a significant increase in symptoms of depression and anxiety from pre- to mid-pandemic as well as a significant increase in the prevalence of eating disorders. **Conclusions**: By late 2020, mental health in the student population had deteriorated compared to pre-pandemic levels. These findings provide evidence for increased levels of depression, anxiety, and eating disorders related to the COVID-19 pandemic. There is a need for better preparedness for future crises in order to mitigate the impact on student mental health.

## 1. Introduction

Mental health among students has become a pressing public concern in recent years. The stressful events associated with the transition to university life leave young people susceptible to symptoms of common mental distress [1]. A comprehensive mental health survey of students attending a large university in northern England revealed high rates of mental distress in 2016, with particularly elevated rates of severe anxiety and depression [2]. Following the onset of the COVID-19 pandemic, mental health was recognised as an area of concern. In particular, the public health measures necessary to prevent the transmission of COVID-19 may have had major effects on student mental health, as this specific population experienced a range of unique stressors, including sudden shifts to remote learning, loss of campus community and support networks, and increased academic pressure and uncertainty around the future.

Early evidence assessing the mental health implications of COVID-19 identified a heightened prevalence of anxiety and depressive symptoms. For instance, a World Health Organisation report found an increase in prevalence of depression and anxiety in the first year of the pandemic [3]. With regard to the student population, a cross-sectional US study found that 71% of students reported increased stress and anxiety due to the COVID-19 pandemic, with fear and reduced social interactions identified as contributors [4], while a study of UAE students found significant increases in anxiety as universities moved to online instruction [5].

Using population-representative surveys in Australia between June 2020 and June 2022, Joyce and colleagues [6] found that although young people reported high levels of psychological distress at each survey point, levels of distress remained stable over time, irrespective of lockdown periods or government restrictions. A number of longitudinal and repeated cross-sectional studies have, however, demonstrated an increase in psychological distress among university students studying in different countries. A UK study using a longitudinal design found a decrease in mental wellbeing in a sample of 255 university students [7], while a repeated cross-sectional study revealed that levels of depression reported by university students from southern Italy during the pandemic significantly increased from those reported in 2017 [8]. Against their predictions, however, Zurlo and colleagues found that the levels of anxiety remained similar. Using a longitudinal design, Evans and colleagues compared data collected in April/May 2020 (during lockdown) to autumn 2019 (baseline, pre-pandemic), and found a decrease in wellbeing and an increase in depressive symptoms in a sample of 254 UK undergraduate students [9]. There was, however, no increase in anxiety levels. Together, the literature suggests that depression rates increased among student samples during the pandemic; however, the findings related to anxiety are equivocal.

Although there are some longitudinal or repeated cross-sectional studies exploring the impact of COVID-19 on common mental health difficulties, less focus has been directed towards the impact of the pandemic on eating disorders. The pandemic introduced a number of stressors, including social isolation, uncertainty, and disrupted routines, which are known to exacerbate or trigger eating disorders. Additionally, eating disorders can be influenced by factors such as body image concerns, academic pressure, and social media exposure, all of which were heightened during the pandemic. One repeated cross-sectional study of French students found the prevalence of eating disorders was stable between 2009 and 2018 but increased sharply in 2021 for both males and females [10]. Thus, it is important to determine the impact of the COVID-19 pandemic on students in relation to the prevalence of eating disorders given that the pseudo-longitudinal research evidence base is limited in relation to this condition. By focusing on eating disorders, our study addresses a critical aspect of student mental health, providing a more comprehensive understanding of the pandemic’s mental health impact.

In sum, while past work has investigated links between pandemic-related factors and common mental health difficulties, little work has focused on less common mental health conditions (e.g., eating disorders). Moreover, as noted above, the research on anxiety is contradictory and may depend on cultural and individual factors. The current study, therefore, aimed to detail the prevalence of mental distress in the student population at an early stage in the COVID-19 pandemic, understand the psychological impact of the COVID-19 pandemic on UK student mental health, and, using a repeated cross-sectional design, compare UK university students’ psychological health conditions before and during the pandemic. We hypothesised that there would be higher levels of (i) depression, (ii) anxiety, and (iii) eating disorders during the pandemic compared to before the pandemic.

## 2. Methods

### 2.1. Ethical Approval

Ethical approval was received from the Institute of Population Health Sciences’ (IPHS) Research Ethics Committee. All participants have given consent for their data to be used in the research.

### 2.2. Design

This study used data from an annual survey that assesses student mental health to explore differences in the data between 2019 (prior to the pandemic) and 2020 (during the pandemic) collected from students attending one of two universities in northern England. Thus, the research design is a repeated cross-sectional design as the two time-points comprise independent samples.

### 2.3. Participants

The current study recruited students attending one of two large universities in northern England. The online Student Mental Health survey was open to all students attending these institutions and the survey link was sent via email in October 2019 and October 2020. At baseline in 2019, 4812 UK university students were sampled. Thirty-three per cent were male and 67% were female. The mean age was 23 years (±5.67), and the majority were from a white ethnic background (81%). In 2020, 3817 students completed the survey. The majority of participants were female (71%). The mean age was 23 years (±5.87), and the majority were from a white ethnic background (85%). Thus, the two samples were very well matched on demographic characteristics.

### 2.4. Measures

#### 2.4.1. Generalised Anxiety Disorder-7 (GAD-7; [11])

The GAD-7 assesses symptoms of anxiety over the last two weeks. Example items include ‘feeling nervous, anxious or on edge’ and ‘not being able to stop or control worrying’. All items are scored on a 4-point scale (0 = *not at all* to 3 = *nearly every day*). Total scores range from 0 to 21, with higher scores indicating greater severity of anxiety. The cut-off points for mild, moderate, and severe levels of anxiety are scores of 5, 10, and 15, respectively. The internal consistency of the GAD-7 was *α* = 0.92 in the 2020 data set and *α* = 0.93 in the 2019 data set.

#### 2.4.2. Patient Health Questionnaire (PHQ-9; [12])

The PHQ-9 assesses symptoms of depression over the last two weeks. Example items include ‘feeling down, depressed, or hopeless’ and ‘thoughts that you would be better off dead’. Responses are recorded on a 4-point scale (0 = *not at all* to 3 = *nearly every day*). Total scores range from 0 to 27, with higher scores suggesting greater severity of depression. The cut-off points for mild, moderate, and moderately severe/severe depression are 5, 10, and 15, respectively. The internal consistency of the PHQ-9 was *α* = 0.90 in the 2020 data set and *α* = 0.91 in the 2019 data set.

#### 2.4.3. Eating Disorders

The participants were asked whether they considered themselves as having an eating disorder (‘Do you consider yourself as having an eating disorder?’). The presence of an eating disorder was coded as 1 (yes) or 0 (no).

### 2.5. Statistical Analysis

A series of Kolmogorov–Smirnov tests were conducted to assess the normality of the distribution for each common mental health condition variable, namely PHQ-9 and GAD-7. The results indicated that all variables showed significant deviations from normality: PHQ-9 in 2019, (*D*(4812) = 0.09, *p* < 0.001); GAD-7 in 2019 (*D*(4812) = 0.10, *p* < 0.001); PHQ-9 in 2020 (*D*(3817) = 0.08, *p* < 0.001); and GAD-7 in 2020 (*D*(3817) = 0.09, *p* < 0.001). Thus, in order to explore differences in university students’ psychological health before and during the pandemic, Mann–Whitney U tests were carried out to compare levels of depression and anxiety during the pandemic with those reported by students at baseline. The data were analysed using a chi-square test for independence to determine whether there was a significant difference in the prevalence of eating disorders between 2019 and 2020.

## 3. Results

### 3.1. Descriptive Analysis

**Figure 1:**
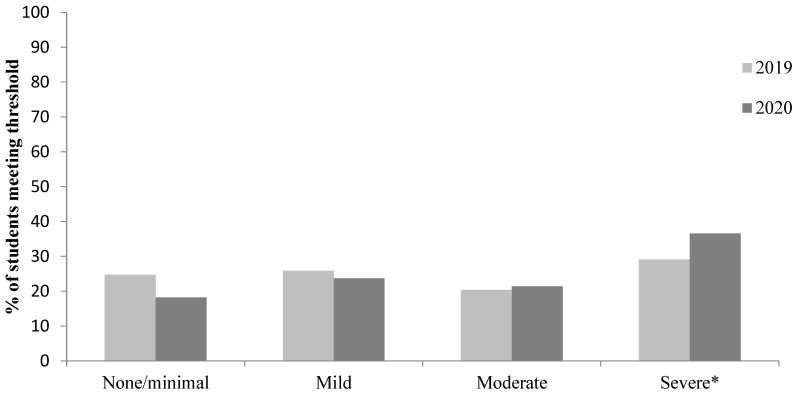
Proportion of students in each sample meeting the criteria for none/minimal (0–4), mild (5–9), moderate (10–14), and moderately severe/severe depression (15–27). * Moderately severe and severe depression.

**Figure 2:**
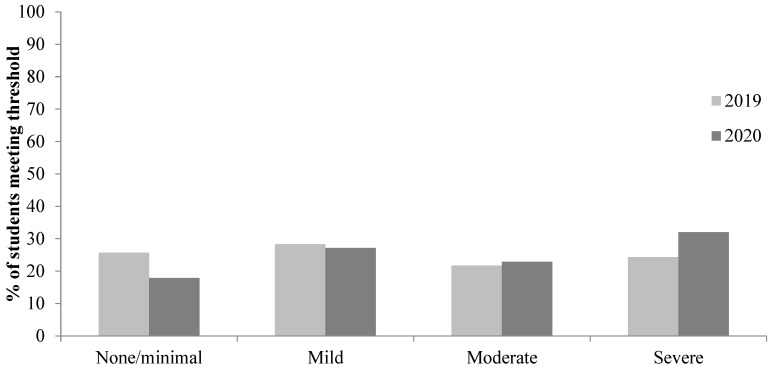
Proportion of students in each sample meeting the criteria for none/minimal (0–4), mild (5–9), moderate (11–14), and severe anxiety (15–21).

As shown in Figure 1 and Figure 2, using the published criteria for moderate anxiety (10–14) and depression (10–14), the proportion of students above these cut-offs was 46.0% for anxiety and 49.5% for depression before the COVID-19 pandemic. The proportion of students above these cut-offs during the COVID-19 pandemic increased to 54.9% for anxiety and 58.1% for depression. Using the published criteria for severe anxiety (GAD-7: 15–21), 24.3% met the criteria for severe anxiety before the COVID-19 pandemic, whereas 32.0% met the criteria for severe anxiety during the COVID-19 pandemic. Using the published criteria for moderately severe and severe depression (PHQ-9: 15–19 moderately severe and 20–27 severe), 29.1% met the criteria for moderately severe/severe depression before the COVID-19 pandemic, whilst 36.6% met the criteria for moderately severe/severe depression during the COVID-19 pandemic.

### 3.2. Depression and Anxiety

A Mann–Whitney test indicated that students experienced higher levels of depression during the COVID-19 pandemic (Mdn = 11, IQR = 12), compared to before the pandemic (Mdn = 9, IQR = 11), *U* = 8,110,583, *p* < 0.001, *r* = −0.10. A Mann–Whitney test indicated that students experienced higher levels of anxiety during the COVID-19 pandemic (Mdn = 11, IQR = 10) compared to before the pandemic (Mdn = 9, IQR = 10), *U* = 7,998,636, *p* < 0.001, *r* = −0.11.

### 3.3. Eating Disorders

A chi-square test for independence was conducted to examine the relationship between time points (pre vs. during-pandemic) and the prevalence of eating disorders. The results indicated a significant association between time points and eating disorder prevalence, χ^2^ (1, N = 8617) = 14.51, *p* < 0.001. The percentage of students reporting an eating disorder in 2019 was 10.2%, whereas in 2020, the percentage increased to 12.8%.

## 4. Discussion

The current study aimed to detail the prevalence of mental distress in the student population at an early stage in the COVID-19 pandemic as well as understand the psychological impact of the pandemic on student mental health by comparing levels of depression, anxiety, and eating disorders before and during the pandemic.

Overall, there were high levels of depression and anxiety in our student samples, especially during the COVID-19 pandemic as nearly a third of students scored above the cut-off for severe anxiety (32%), whilst just over a third of students scored above the cut-off for moderately severe depression (36.6%). The number of students in the scoring above the cut-off for severe anxiety and moderately severe depression is higher than those recorded in our previous research, as we found that 20.9% met the criteria for severe anxiety and 11.3% met the criteria for severe depression in October 2016 (see [2]). This suggests a substantial increase in depression since 2016, indicating an upward trend that predates the COVID-19 pandemic. Also, as one in six people report experiencing a common mental health problem in a given week in England [13], the prevalence of common mental health difficulties was higher in our student sample during the early stages of the pandemic.

Consistent with research exploring mental health among the general population before and during the COVID-19 pandemic (e.g., [3,14]), students reported higher levels of depression and anxiety during the COVID-19 pandemic. Although we found an increase in prevalence of anxiety during the first year of the pandemic, this finding conflicts with findings from Zurlo et al. [8] and Evans et al. [9]. Nevertheless, our findings partially support those reported by Zurlo and colleagues [8] who found an increase in symptoms of depression among students from southern Italy. One explanation for the increase in common mental health difficulties among students could be social isolation resulting from the restrictions imposed in response to the pandemic [15]. Restrictions on gatherings and social events curtailed students’ ability to engage in extracurricular activities. Alongside the absence of regular campus routines, the lack of in-person interaction with lecturers and peers may have contributed to feelings of distress. Nevertheless, although social isolation is generally associated with poorer mental health outcomes, research conducted during the pandemic has been mixed [6]. As a result, the relationship between social isolation and mental health during this period remains inconclusive, suggesting that other contributing factors may also be important (see [15]). For example, intolerance of uncertainty has been cited as a stressor leading to common mental health difficulties, particularly anxiety [16]. Due to the unpredictable nature of the pandemic, students who struggled with uncertainty may have found it difficult to cope, leading to feelings of depression and anxiety [15]. Thus, the elevated rates of mental distress among students may be attributable to increased social isolation, along with other contributing factors such as financial stress, academic disruption, and intolerance of uncertainty.

Consistent with previous research (e.g., [10]), the results of this study indicate a significant increase in the prevalence of eating disorders from 2019 to 2020. Many stressors that are considered risk factors for eating disorders, such as food insecurity and disruption in routine, were exacerbated during the pandemic [17]. Increased use of social media during periods of lockdown could have also contributed to the significant increase in eating disorders, as these platforms often exacerbate factors that contribute to these conditions [18]. In particular, Rodgers and colleagues noted that social restrictions may have increased exposure to harmful eating- and appearance-related images on social media [19]. However, this finding should be treated with caution due to the inclusion of a single item for the presence of an eating disorder.

Given the findings of our study, which illustrate that young adults studying at university may be particularly prone to experiencing high levels of mental distress during a pandemic, there are several recommendations that could enhance emergency preparedness and support student wellbeing during future crises to mitigate the impact on student mental health. For instance, higher education institutions could incorporate mental health education into curricula while also developing comprehensive emergency mental health plans that prioritise online support. In addition to this, it is essential for governments to increase funding for university mental health services, ensuring adequate resources for support, particularly via remote means. Together, these recommendations will create a more resilient and responsive mental health infrastructure for students in times of crisis.

Our findings should, however, be viewed in light of several limitations. As the sample comprised students attending one of two universities in northern England, our findings may not be generalisable. Our participants may represent a specific subset of the population as they were all self-selected volunteers. It is possible that there was a bias in our sample, as those experiencing mental health difficulties at the time the survey was launched may have been more likely to be attracted by the survey advertisement. Our sample is therefore not representative of the larger population of students. The high proportion of females suggests that our samples may not fully representative the entire student population. Finally, as we used a repeated cross-sectional design, the findings may be influenced by sample differences. However, the samples were very well matched on demographic characteristics. As these data were collected in the early stages of the pandemic, future research should continue to assess mental health as the crisis continues to unfold. While this question has been examined longitudinally in vulnerable populations such as young people [8], future research could test the idea longitudinally, specifically examining university students. In particular, future longitudinal research could explore the role of psychological and social mechanisms, such as intolerance of uncertainty, perceptions of control, and/or loneliness, especially as the relationship between isolation and mental health remains inconclusive in this context. Further to this, given the increase in symptoms of depression and anxiety, future research could implement and test interventions aimed at mitigating these effects during other stressful periods, such as exams.

## 5. Conclusions

In conclusion, the mental health and wellbeing of the student population appears to have been affected by the COVID-19 pandemic. In particular, this study identifies high levels of depression and anxiety among students during the early stages of the COVID-19 pandemic. As these findings illustrate that young adults studying at university may be particularly prone to experiencing high levels of depression and anxiety during a pandemic, there is a need for better preparedness for future crises in order to mitigate the impact on student mental health. It is possible that the pandemic may have long-lasting effects, with some students potentially developing chronic mental health conditions. Thus, the provision of support to reduce the likelihood of longer-term problems would be beneficial.

## Data Availability

The original contributions presented in the study are included in the article. Further inquiries can be directed to the corresponding author.
